# A chimeric EBV gp350/220-based VLP replicates the virion B-cell attachment mechanism and elicits long-lasting neutralizing antibodies in mice

**DOI:** 10.1186/s12967-015-0415-2

**Published:** 2015-02-06

**Authors:** Javier Gordon Ogembo, Matthew R Muraswki, Lori W McGinnes, Agapi Parcharidou, Rujapak Sutiwisesak, Timelia Tison, Juan Avendano, Deep Agnani, Robert W Finberg, Trudy G Morrison, Joyce D Fingeroth

**Affiliations:** Department of Medicine, University of Massachusetts Medical School, 364 Plantation Street, LRB Room 323, Worcester, MA 01605 USA; Department of Medicine, Beth Israel Deaconess Medical Center/Harvard Medical School, Boston, MA UK; Department of Microbiology and Physiological Systems, University of Massachusetts Medical School, Worcester, MA USA; Program in Immunology and Microbiology, University of Massachusetts Medical School, Worcester, MA USA

**Keywords:** EBV, NDV, VLP, Vaccine, Neutralization

## Abstract

Epstein-Barr virus (EBV), an oncogenic gammaherpesvirus, causes acute infectious mononucleosis (AIM) and is linked to the development of several human malignancies. There is an urgent need for a vaccine that is safe, prevents infection and/or limits disease. Unique among human herpesviruses, glycoprotein (gp)350/220, which initiates EBV attachment to susceptible host cells, is the major ligand on the EBV envelope and is highly conserved. Interaction between gp350/220 and complement receptor type 2 (CR2)/CD21 and/or (CR1)/CD35 on B-cells is required for infection. Potent antibody responses to gp350/220 occur in animal models and humans. Thus, gp350/220 provides an attractive candidate for prophylactic subunit vaccine development. However, in a recent Phase II clinical trial immunization with soluble recombinant gp350 reduced the incidence of AIM, but did not prevent infection. Despite various attempts to produce an EBV vaccine, no vaccine is licensed. Herein we describe a sub-unit vaccine against EBV based on a novel Newcastle disease virus (NDV)-virus-like particle (VLP) platform consisting of EBVgp350/220 ectodomain fused to NDV-fusion (F) protein. The chimeric protein EBVgp350/220-F is incorporated into the membrane of a VLP composed of the NDV matrix and nucleoprotein. The particles resemble native EBV in diameter and shape and bind CD21 and CD35. Immunization of BALB/c mice with EBVgp350/220-F VLPs elicited strong, long-lasting neutralizing antibody responses when assessed *in vitro*. This chimeric VLP is predicted to provide a superior safety profile as it is efficiently produced in Chinese hamster ovary (CHO) cells using a platform devoid of human nucleic acid and EBV-transforming genes.

## Introduction

Epstein-Barr virus (EBV) is an oncogenic human herpesvirus [1]. By adulthood, EBV has infected >95% of the global population. Like all herpesviruses, EBV persists for life due to a complex life cycle consisting of alternate latent and lytic phases [1]. In the developing world, primary EBV infection typically occurs during early childhood and is asymptomatic. However, in malaria endemic regions childhood acquisition poses a heightened risk of EBV+ Burkitt’s lymphoma [1,2]. In high income countries primary infection is often delayed [2] resulting in a syndrome known as acute infectious mononucleosis (AIM) among 50-77% of adolescents/young adults [3,4]. Although AIM is normally self-limited, illness can be prolonged. Catastrophic sequelae such as splenic rupture and hemophagocytosis may be life threatening, and AIM increases the risk of developing EBV+ Hodgkin lymphoma [1,2]. Consistent with its epithelial tropism, EBV is also highly associated with nasopharyngeal carcinoma and certain gastric and sporadic carcinomas [1]. Among healthy individuals, lytic EBV replication is limited and the pool of latently infected cells remains stable [5]. However, under conditions of immunosuppression, replication can accelerate, leading to expansion of EBV-infected cells. This increases the likelihood of de novo transformation of B-cells as seen in EBV+ post-transplant lymphoproliferative disorders (PTLD) and HIV-associated B-cell lymphomas [6,7]. Uninfected solid organ and stem cell transplant recipients are particularly susceptible to developing EBV+ PTLD in settings where preexisting EBV-specific immunity is lacking [8,9]. To date, disease management is suboptimal with frequent relapses and high treatment-related mortality.

Because of its oncogenic potential, options for developing safe prophylactic and/or therapeutic EBV vaccines have been limited. The recent success of subunit-based virus-like particle (VLP) vaccines targeting hepatitis B and human papillomavirus and their associated tumors [10,11], suggests a similar strategy could prove successful for EBV. EBV is distinct from other herpesviruses, as a single antigen, glycoprotein (gp)350/220, is the predominant virion envelope protein [1,12]. Interactions between EBVgp350/220 and complement receptor type 2 (CR2)/CD21 and/or (CR1)/CD35 on B-cells is required for cellular attachment and initiation of latent infection [13-15]. Therefore, gp350/220 is an attractive candidate for development of a prophylactic subunit vaccine.

Pre-existing antibodies provide a first line of defense against many viral pathogens. Much evidence indicates that neutralizing antibodies targeting gp350/220 are present in humans, prevent neonatal infection, and are readily generated in response to immunization of humans and other animals [16]. Nevertheless, in a Phase I/II clinical trial in young adults, vaccination with purified soluble recombinant gp350/220 did not prevent infection, although AIM was reduced [17]. While these observations indicate immunity to gp350/220 can limit clinical disease, the variable success of recombinant soluble protein trials underscore the historical observation that soluble protein vaccines are generally limited in immunogenicity [18] and do not elicit long-term protection. More physiologic presentation of gp350/220 to the immune system as would occur on exposure to repetitive units of gp350/220 mimicking the virion envelope, together with development of a vehicle such as a VLP with potential for recruiting innate immune cells [19] that robustly stimulate antibody production might achieve this goal.

In this study, we utilized a novel Newcastle disease virus (NDV)-based VLP as a delivery platform to display the gp350/220 ectodomain (ED) in particulate form. Typical VLPs assemble from one or more viral structural proteins forming repetitive arrays that resemble native virus – but usually lack viral nucleic acid [20]. Herein we report generation of a chimeric EBV-VLP based on gp350/220 and the NDV-fusion (F) protein (EBVgp350/220-F). These VLPs are distinct from recently described complex EBV-VLPs that require assembly in immortal human cell lines [21], may contain viral DNA/RNA and are inefficiently produced and released [22]. The chimeric EBVgp350/220-F VLPs are similar in diameter and shape to EBV virions and present repeated arrays of gp350/220 on a membranous surface. They are efficiently produced and released from non-human cell types such as Chinese hamster ovary (CHO), a Federal Drug Agency (FDA) approved vehicle. Immunization of BALB/c mice with EBVgp350/220-F VLPs without adjuvant elicited strong and long lasting neutralizing antibody responses able to block EBV infection *in vitro*.

## Materials and methods

### Virus

B95-8 strain EBfaV-GFP was provided by Richard Longnecker (Northwestern University, Chicago). EGFP-EBV was prepared as described [23]. Kaposi’s sarcoma herpesvirus (KSHV) and EBV-EGFP from the AGS line were gifts from Christine King (SUNY Upstate Medical School) and Liisa Selin (University of Massachusetts Medical School), respectively.

### Cell lines

293A, a clone of 293 (human embryonic kidney) was from Life Science Technologies. 293 T, a 293 derivative expressing SV-40 T antigen, CHO, Vero (African green monkey kidney), ELL-0 (chicken embryo), K562 (human erythroleukemia), Raji (EBV+ Burkitt’s lymphoma), HB168 (72A1 murine hybridoma) were from the American Type Culture Collection. 293A, CHO, and ELL-0 were cultured in DMEM. Other lines were grown in RPMI media. All media contained 1% L-glutamine, 10% heat-inactivated fetal bovine serum (FBS) and 2% penicillin-streptomycin.

### Plasmid vectors

Full length EBVgp350/220 (BLLF1) isolated from the BamHI L fragment of B95-8 [24] by PCR was cloned into pCAGGS [25]. An upstream primer containing a SalI restriction enzyme site and a Kozak sequence: Forward Primer: 5′- AAC ATA GCT GAC GCC ACC ATG GAG GCA GCC TTG CT -3′ together with a downstream primer that incorporated a double stop followed by a NruI site: Reverse Primer: 5′- TTG ATA TCG CGA CTA TTA TTA TAC ATA GGT CTC GG -3′ was used. After amplification, the PCR product was digested with SalI and NruI, purified and ligated into pCAGGS pre-digested with XhoI and MscI. Fidelity was confirmed by DNA sequencing.

### pCAGGS-EBVgp350/220-F

A chimeric fragment consisting of nucleotides encoding gp350/220 ectodomain (ED), amino acids 1–864, fused to the NDV-F heptad repeat 2 (HR2), transmembrane (TM) and cytoplasmic (CT) domains, amino acids 466–553, was constructed by three-way ligation (EBVgp350/220-F). A PCR product containing the gp350/220 ED was digested with SalI and ScaI. A PCR fragment encoding the NDV-F membrane proximal HR2 domain, TM and CT domains in addition to adjacent pCAGGS vector sequences was PCR amplified from pCAGGS-F [26] incorporating a ScaI site upstream and a HindIII site downstream. Forward Primer: 5′- AAC ATA AGT ACT GCT TGG GAA TGT CAA CAA CTC G -3′ and Reverse Primer: 5′- TAC GCC AAG CTT GGG CTG CAG GTC GAG GGA TCT CCA -3′. This isolated F fragment was cut with ScaI and HindIII. The pCAGGS vector was digested with XhoI and HindIII and gel-purified. The three respective DNA fragments were co-incubated in a 5:5:1 ratio with T4 DNA ligase generating pCAGGS-EBVgp350/220-F. Fidelity was confirmed by DNA sequencing. pCAGGS-F, pCAGGS-M and pCAGGS-NP derived from NDV (Avulavirus) have been described [26].

### Transfection, generation and purification of gp350/220-F VLPs

1.0 μg/well of pCAGGS, pCAGGS-gp350/220 wildtype (WT) or pCAGGS-EBVgp350/220-F were individually transfected into 80% subconfluent CHO, 293A, Vero and ELL-0 cells seeded in six-well tissue culture plates using Lipofectamine and Reagent Plus (Life Sciences Technologies) according to the manufacturer’s direction. Cells were harvested at 48 h post-transfection to assess surface expression of gp350/220 by cytometry.

Small-scale and large-scale VLP stocks were prepared as outlined [27]. For large-scale preparation, equal amounts (8 μg/plasmid) of pCAGGS-NDV-M, −NP and pCAGGS-EBVgp350/220-F plasmids were co-transfected into cells seeded in T-175 cm^2^ flasks. DNA-Lipofectamine complexes were incubated at 37°C for 5 h with 293 T and ELL-0 or overnight with CHO cells. Complexes were removed by washing and 20 ml of complete media was added. For some preparations 20 ml of complete media containing 4 mM of sodium butyrate (Millipore ED) and 20 ng/ml of TPA (12-O-tetradecanonylphorbol 13-acetate) (Sigma) was added as chemical induction had been reported to increase production of HIV VLPs [28], but the above addition did not significantly alter VLP yield in our hands. Eleven flasks were seeded for each large-scale preparation. VLPs were isolated by sedimentation and sucrose gradient purification [27].

### Antibodies

Primary: Monoclonal antibodies (mAb)-72A1 and -2 L10 anti-gp350/220 were from EMD Millipore. Polyclonal rabbit anti-NDV and anti-HR2 have been described [29]. MAb anti-CD35 (clone E11) and anti-CD21 (clone LT21) were from BioLegend.

Secondary: Horseradish peroxidase (HRP)-conjugated goat anti-mouse IgG (total and isotype specific) antibodies or goat anti-rabbit antibodies for immunoblot or ELISA were from Sigma. Goat F(ab’)2 anti-mouse IgG (H + L) AF488 or AF594 was used for cytometric and confocal analyses (Invitrogen). Goat anti-mouse IgG (H + L) immunogold was used for electron microscopy (EM) (Aurion).

### Biochemical analysis

Purified EBV, VLPs or cells were lysed in RIPA buffer (Boston Bioproducts) containing a complete protease inhibitor cocktail (Roche Applied Science). Lysates were incubated for 15 min on ice, microcentrifuged for 5 min at 10,000 rpm and the protein content of each lysate determined by Bradford assay. Lysates were boiled for 5 min in Laemmli SDS-sample buffer (Boston Bioproducts) under non-reducing conditions. 10 μg of lysate was loaded onto a 4–12% polyacrylamide gel for protein separation. Protein bands were detected by Pierce’s silver stain kit according to the manufacturer’s recommendation. For immunoblot, proteins were transferred to a PVDF membrane using iblot (Life Sciences Tech.). Membranes were blocked with 5% non-fat dry milk (LabScientific) for 30 min and detected with specific antibodies as described in Results.

### Immunogold analysis (EM)

VLPs and viruses were analyzed by EM as described [30]. Purified particles were dialyzed against 1 L of TNE (100 mM Tris; 2.0 M NaCl; 10 mM EDTA; pH 7.4) to remove residual sucrose, incubated with 3% bovine serum albumin (BSA) in TNE for 45 min. and embedded on a grid. A 1:40 dilution of mAb-72A1 (10 ug/ml) in 1% BSA/TNE was added to the grid for 1 h at RT, washed and the grid re-incubated with gold-conjugated anti-mouse IgG for 1 h. After two final washes, the grid was negatively stained with 12% phosphotungstic acid (pH 7) for 15 sec, air dried for 30 min and examined using a Tecnai transmission electron microscope (FEI).

### Binding assays, confocal microscopy and cytometry

Confocal microscopy was performed as outlined [14]. 10^6^ cells seeded onto Labtek slides were incubated at RT for 1 h with uniform amounts of VLP pre-determined by silver stain and/or Bradford assay. Mixtures of cells and VLPs were first stained with anti-CD21, anti-CD35 or isotype control for 30 min on ice, washed 2x and re-incubated with AF594-goat anti-mouse antibodies, washed and further stained with AF488-coupled-mAb-2 L10 for 30 min on ice. Nuclei were stained with DAPI 33342 (Sigma) for 5 min at RT. Cells were finally washed 2x, mounted with Mounting Medium (DakoCytomation) and imaged with an UPlanApo 60x1.42 NA objective on an Olympus BX62 microscope fitted with a cooled Hamamatsu Orca AG CCD camera. Microscope, filters, and camera were operated as outlined [31]. Deconvolution was as described [14].

To analyze cell surface gp350/220 expression, cells transfected with relevant plasmids were suspended in PBS 2% heat-inactivated FBS, washed twice with PBS, incubated with anti-gp350/220 (mAb-72A1), washed twice, re-incubated with goat anti-mouse-AF488 and re-washed. Cytometric analysis was performed on a FACScan or LSRII benchtop FC (Becton-Dickinson). Data were analyzed using CellQuest Pro Version 4.0.1 (B-D) and/or FlowJo Cytometry software (Tree Star Inc). A minimum 10,000 events was recorded for each analysis. Experiments were repeated 3x.

### Immunization

Groups of five 6–8 week BALB/c mice (Jackson Laboratories) were immunized intraperitoneally with 10 μg EBVgp350/220-F VLP, soluble recombinant EBVgp350/220 ED protein (amino acids 4–863; Immune Technology) (positive control), or UV-inactivated EGFP-EBV (UV-EBV) (positive control). UV-inactivation was achieved by exposure to 254 nm UV light for 5 min (source model UVG-11, UVP) from a distance of 10 cm, producing complete loss of EGFP expression upon EBV-superinfection of Raji (see below). Gp350/220 content of the respective immunogens was normalized by silver stain. Immunogens were re-suspended to a final volume of 0.5 ml in TNE 10% sucrose or in 10% sucrose TNE buffer alone (vehicle control). Mice were boosted with equal amounts of antigen on days 43, 172, 183 and 218. After primary immunization, tail vein serum was obtained at two-week intervals until day 84, then again on days 154, 186, and 197. A terminal bleed was performed on day 228. Long-term antibody titers and neutralization were first assessed on day 56 to optimize the neutralization assay (data not shown). Thereafter neutralization was assessed with serum obtained at day 154 or 111 days after a single boost on day 43. Subsequently, three additional immunizations were administered on days 172, 183 and 218. All time points were used to determine anti-gp350/220 specific IgGs. Following the final boost mice were sacrificed on day 228 to again obtain sera for determination of anti-gp350/220 neutralization assays that followed a four-boost regimen. TNE served as a negative control. Experiments were repeated at least twice. Animal procedures were performed in accordance with the University of Massachusetts Medical School IACUC and Institutional Biosafety Committee.

### Enzyme-linked Immunosorbent Assay (ELISA)

IgG titers were measured by ELISA [32] using soluble gp350/220 ED as target antigen. Briefly, 96-well microtiter plates (Nunc-Immuno Plate Maxisorp) were coated with 50 ng/well of recombinant gp350/220 ED in a carbonate buffer (pH 6.2) at 4° C overnight and blocked with 1% BSA. Serially diluted sera in PBS was added for 2 h at RT and washed. Antibody binding was detected with HRP-labeled goat anti-mouse IgG, IgG1, IgG2a, IgG2b and IgG3 secondary antibodies at RT for 1 h. Plates were washed 5x and the substrate tetramethylbenzidine (Life Science Technologies) was added. Reactions were stopped with 2 M sulfuric acid. To determine antibody titer, optical density was read at 450 nm with an ELISA reader (Spectramax® Plus 384, Molecular Devices). The highest antibody dilution yielding an OD_450_ 2x higher than that of TNE-treated mice was designated the endpoint titer. Anti-gp350/220 mAbs served as positive controls.

### Neutralization studies

Day 154 sera from mice that had received a single booster on day 43 and day 228 sera (terminal) from mice serially immunized with EBVgp350/220-F VLP, UV-EBV, soluble gp350/220 ED and TNE were heat-inactivated at 56°C to remove complement. Sera were then diluted as described and then pre-incubated 1:1 with purified EGFP-EBV at different concentrations (typically 5 or 10 microliters of a 1:5 virus dilution from a single frozen stock) for 1 h at RT before infecting 10^5^ Raji cells seeded in 24-well tissue culture plates as previously outlined [23,33]. Anti-gp350/220 mAb-72A1 (neutralizing), mAb-2 L10 (non-neutralizing) and sera from TNE-treated mice (vehicle) served as controls. Experiments were repeated at least 3 times. Plates were incubated at 37°C for 3 days and visualized daily to enumerate GFP^+^ cells. Cytometry was performed on day 3 for end-point analysis.

### Statistical analysis

Neutralization data were analyzed using Graph Pad Prism 6 Software (GraphPad Software, Inc., San Diego). The difference between neutralizing antibody titers of immunized and non-immunized (TNE-vehicle control) mice was determined using the unpaired two-tailed *t* test for independent groups. Statistical significance of the tests was based on two-sided p values ≤ 0.05.

## Results

### Detection of chimeric EBV-gp350/220-F on the surface of mammalian and avian cell lines

**Figure 1:**
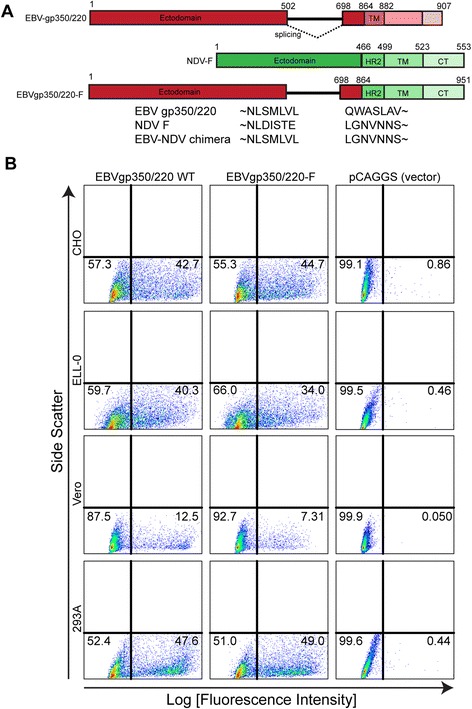
**Construction and expression of EBVgp350/220-F chimeric protein. (A)** Diagram of full length EBVgp350/220-WT (top), full length wild type NDV-F (middle), and chimeric EBVgp350/220-F (bottom) (not to scale). C-terminal amino acid sequences comprising the gp350/220 ectodomain (ED) and N-terminal sequences from NDV-F HR2 at the point of fusion are indicated. The bolded black line represents amino acid sequences deleted in frame in the gp220 isoform. Both isoforms contain the N-terminal B-cell attachment epitope. **(B)** Expression of EBVgp350/220-WT and EBVgp350/220-F on the surface of four cell lines (CHO, ELL-0, Vero and 293A). 10^6^ cells from each line were transfected with 1 μg of pCAGGS-EBVgp350/220 WT, pCAGGS-EBVgp350/220-F or pCAGGS alone (vector control). At 72 h post-transfection, cells were stained with anti-gp350/220 mAb-72A1 followed by AF488-coupled goat anti-mouse IgG (H + L) and analyzed by flow cytometry.

VLPs assemble from three or four virion proteins, the envelope glycoproteins, F (fusion, a type 1 glycoprotein) and/or HN (hemaglutinin-neuraminidase, a type 2 glycoprotein), together with core proteins M (matrix) and NP (nucleocapsid protein) that drive assembly and release [27,34]. Recently it was demonstrated that the ability to form VLPs is preserved when the ED of F is substituted with an ED derived from unrelated viral envelope type 1 proteins as long as the TM and CT of F remain intact [35]. To generate EBVgp350/220 bearing VLPs, we first constructed an EBVgp350/220-F chimeric plasmid by fusing the nucleotide sequences encoding amino acids that comprise the entire ED of EBVgp350/220 to sequences encoding NDV-F HR2, TM and CT (Figure 1A). To assess whether the expressed chimera appropriately localized to the plasma membrane, pCAGGS-EBVgp350/220-F and pCAGGS-EBVgp350/220 WT plasmids were individually transfected into cell lines from four different species (CHO, ELL-0, Vero and 293A). Membrane expression of the gp350/220 ED was analyzed by cytometry. MAb-72A1 directed to the N-terminal attachment epitope of gp350/220 [36] detected expression of both gp350/220 WT and chimeric EBVgp350/220-F proteins at the surface of all relevant transfectants (Figure 1B). Anti-gp350/220 mAb-2 L10 [36] that recognizes a distinct epitope likewise detected gp350/220 surface expression (not shown). Comparative expression of WT gp350/220 and chimeric EBVgp350/220-F, within each transfected line, did not significantly differ. Gp350/220 was not detected in cells transfected with an empty pCAGGS vector (negative control).

### Assembly and characterization of chimeric EBVgp350/220-F VLPs

**Figure 2:**
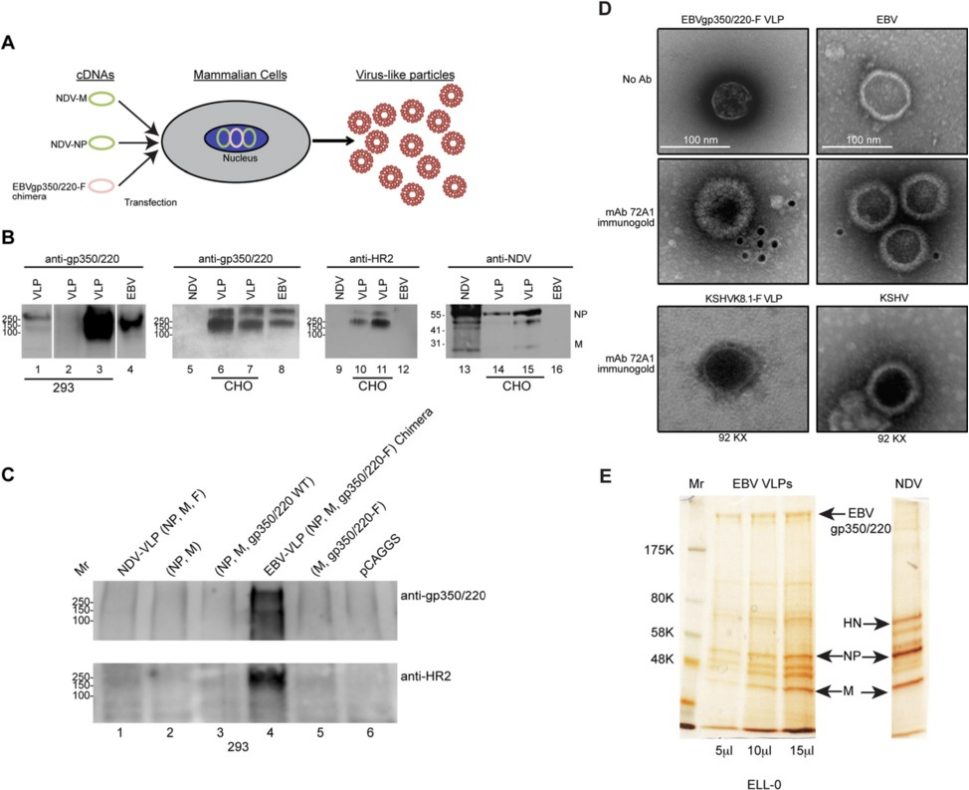
**Assembly and characterization of EBV-gp350/220-F VLPs. (A)** Schematic diagram showing cDNAs pCAGGS-NDV-M, −NDV-NP and -EBVgp350/220-F co-transfected into cells followed by VLP assembly and release. **(B)** Supernatants containing independent EBV-VLP preparations were harvested daily between 24–96 h, concentrated and purified by sucrose-gradient centrifugation followed by particle lysis and immunoblot. mAb-72A1 anti-gp350/220 (left two panels), polyclonal anti-HR2 (third panel) and polyclonal anti-NDV (right panel) detected EBVgp350/220 ED and NDV-F C-terminal peptides respectively. Anti-NDV also detected M and NP in VLPs and NDV, but not EBV. Lanes 1–3 contain VLPs derived from 293T, lanes 6,7,10,11,14, 15 from CHO cells. Lysates of purified EBV (lanes 4, 8, 12, 16) and NDV (lanes 5, 9, 13) were controls. **(C)** Different combinations of pCAGGS plasmids encoding F, M, NP, gp350/220 and gp350/220-F as indicated were co-transfected into 293T cells. Supernatant content was pelleted and analyzed by immunoblot using anti-gp350/220 and anti-HR2 antibodies. Lane 1: NDV-VLP (NP, M, F), lane 2: NP, M, lane 3: NP, M, gp350/220 WT, lane 4: EBV-VLP (NP, M, EBVgp350/220-F), lane 5: M, EBVgp350/220-F, lane 6: pCAGGS . Both antibodies in lane 4 alone detected bands of the expected MW, indicating a chimeric EBV-VLP was specifically assembled and released. **(D)** Electron micrograph of negatively stained sucrose gradient purified EBVgp350/220-F VLPs prepared in CHO cells compared with native EBV -/+ immunogold-coupled antibodies. Top, structure and size of the chimeric VLP compared with EBV without antibody. Middle, demonstration that mAb-72A1 detected by immunogold-coupled goat anti-mouse IgG binds the surface of a chimeric EBVgp350/220-F VLP and EBV (control). Bottom, but not a chimeric KSHV-VLP or KSHV. **(E)** Silver stain of increasing amounts of purified chimeric VLPs released from ELL-0 cells compared with NDV. Positions of EBVgp350/220-F, NDV-NP and -M are indicated by arrows. MW markers are at left.

Following confirmation of plasma membrane expression, as required for particle assembly, pCAGGS-EBVgp350/220-F was co-transfected with NDV core proteins M and NP into different cell lines to generate chimeric VLPs as diagramed in Figure 2A. Particles from distinct preparations released into cell supernatants were purified as outlined [27], lysed and protein analyzed by immunoblot using either anti-gp350/220 (mAb-72A1), polyclonal rabbit anti-NDV HR2 or anti-NDV (entire virus) (Figure 2B) for detection. To confirm the specificity of chimeric VLP formation and antibody recognition, a series of control experiments were performed to document that classical requirements for NDV-VLP assembly were met. Different combinations of pCAGGS plasmids encoding NDV-F, −M, −NP, EBVgp350/220-WT, EBVgp350/220-F as shown in Figure 2C were co-transfected into 293 T cells and the putative particles purified as previously described followed by immunoblot (Figure 2C). Chimeric VLPs expressing EBVgp350/220-F were detected with anti-gp350/220 (top panel) antibody only in cells co-transfected with EBVgp350/220-F, −M and -NP (lane 4), but not in cells transfected with F-M-NP (lane 1), M-NP (lane 2), EBVgp350/220-WT, −M-NP (lane 3), EBVgp350/220-F and -M (lane 5) or pCAGGS alone (lane 6). Anti-HR2 antibody detected the assembled chimeric VLP in lane 4 as well as a WT NDV-VLP in lane 1 (not shown) demonstrating that criteria for VLP formation were satisfied.

To further determine whether incorporation of EBVgp350/220-F into VLPs led to formation of particles similar in size and overall shape to the native virus, purified VLPs were compared to EBV by negative staining and immunogold-conjugated antibody analysis. Figure 2D top shows that purified VLPs containing EBVgp350/220-F were approximately 100 nm in diameter, similar to the diameter of typical EBV virion membranes, though they were less uniform in shape [37]. MAb anti-gp350/220 (72A1) detected with gold conjugated anti-mouse Ig bound the outer surface of both native EBV and the chimeric VLPs, indicating gp350/220 was expressed on the respective envelopes (Figure 2D, middle). Neither mAb-72A1 nor the gold-conjugated secondary antibody bound a KSHVK8.1-derived VLP (J.G.O. manuscript in preparation) or the KSHV envelope (Figure 2D, bottom) confirming the specificity of antibody recognition. The purity of the chimeric VLP compared with NDV and the uniformity of EBVgp350/220-F protein expression on chimeric VLPs was independently analyzed by silver stain (Figure 2E) alternatively revealing the content of these particles.

### Visualization of EBVgp350/220-F VLP attachment to CD21 and CD35 bearing cells

**Figure 3:**
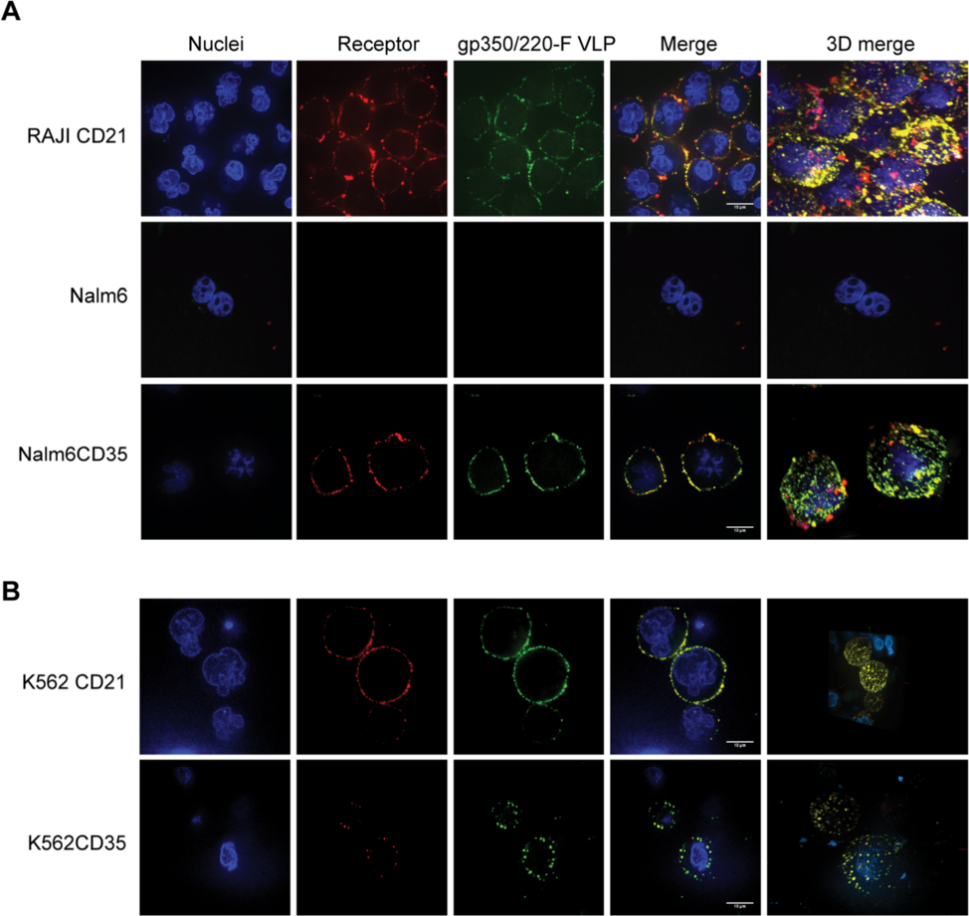
**Purified EBVgp350/220-F VLPs bind CD21 and CD35. (A)** Raji (positive control), Nalm6 (negative control) and CD21 or CD35 transfected sublines of **(A)** Nalm6 and **(B)** K562 were characterized and prepared as described [14]. First, the respective complement receptors were stained with primary mAbs followed by AF594-coupled goat Fab’2 anti-mouse IgG (red). Attachment of EBVgp350/220-F VLPs was next detected directly with AF488-coupled anti-gp350/220 (mAb-2 L10)(green) that recognizes an epitope distal to the attachment site. Cell content was documented by nuclear staining with DAPI. Sequential confocal images showed that the chimeric VLP binds to CD21 or CD35 bearing cells whereas no binding to receptor negative Nalm6 cells was seen. Visualization of 3D merged images confirmed extensive co-localization (yellow) of the chimeric VLPs with both CD21 and CD35.

**Figure 4:**
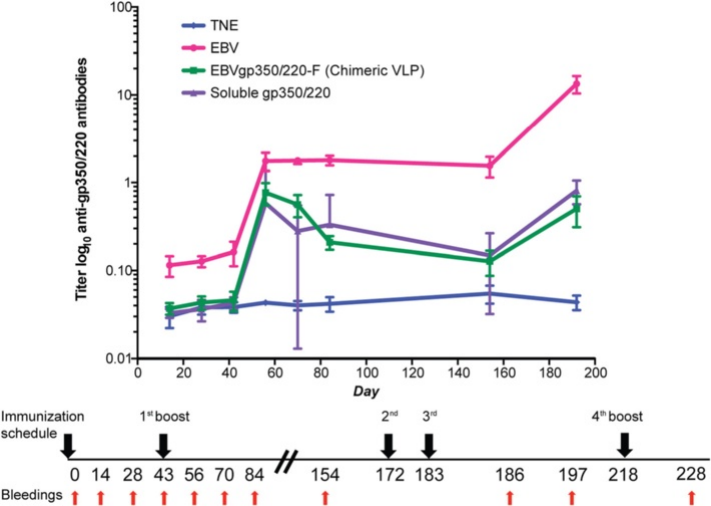
**Long-term IgG anti-gp350/220 antibodies are generated in mice immunized with EBVgp350/220-F VLPs, UV-EBV and soluble recombinant gp350/220 ED.** Groups of five mice were immunized intraperitoneally with either EBVgp350/220-F VLPs (green line), UV-EBV (red line) or soluble recombinant gp350/220 ED (purple line). Each immunogen contained equivalent amounts of gp350/220. TNE served as vehicle control (blue line). All immunizations were performed in the absence of adjuvant. Anti-gp350/220 IgG titers were determined over time up to day 228. Booster immunizations were performed on days 43, 172, 183 and 218 as indicated on the time line (bottom). Primary and booster vaccinations contained equivalent immunogen. Specific anti-gp350/220 ED titers were determined by ELISA (Methods). X-axis shows the day on which serum was obtained for ELISA. Y-axis displays anti-gp350/220 antibody titer on a log scale. Vertical lines indicate the average titer produced by groups of five mice at the time of serum collection and associated error bars indicate the standard deviation.

EBVgp350/220 binds CD21 and/or CD35 on human cells [13,14]. To determine whether chimeric VLPs expressed from CHO cells retained the receptor-binding specificity of the virion envelope protein, we incubated EBVgp350/220-F VLPs with Raji, a latently EBV-infected B-cell line that naturally expresses high amounts of CD21 [13] and can be superinfected with EBV. In addition a panel of receptor negative cell lines, Nalm6 and K562 (not shown), together with their CD21 or CD35 transfectant sublines was investigated [14]. Receptors (red) and VLPs (green) were visualized by indirect immunofluorescence using a confocal microscope for detection. As shown in Figure 3, whereas chimeric VLPs abundantly bound Raji, no attachment to Nalm6 was detected. Nalm6CD21, Nalm6CD35, K562CD21 and K562CD35 all bound EBVgp350/220-F VLPs. Moreover, in each of the receptor bearing sublines, the chimeric VLPs (green) co-localized (yellow) with the relevant cell surface receptor (Figure 4, two right columns).

### Development of specific IgG responses to EBVgp350/220 in BALB/c mice immunized with EBVgp350/220-F VLPs

To determine whether chimeric VLPs elicit EBVgp350/220 specific antibody responses, mice were immunized intraperitoneally with 10 μg of EBVgp350/220-F VLP derived from CHO cells. UV-EBV and soluble gp350/220 ED served as positive controls and TNE as vehicle control. Equivalence of gp350/220 protein content among the different immunogens was determined by silver stain (not shown). All animals received booster immunizations on days 43, 172, 183 and 218. Sera were collected two weeks post-boost. None of the animals displayed signs of local or systemic inflammation or changes in feeding or body weight that would indicate toxicity. Soluble recombinant gp350/220 ED served as the target antigen in an IgG ELISA (Methods). Anti-gp350/220 specific total IgG antibody titers significantly increased among mice immunized with the chimeric VLP, UV-EBV and soluble recombinant gp350/220 ED compared with pre-vaccination and control titers (Figure 4). Historical controls using NDV-F VLPs as immunogen were non-reactive in gp350/220-based ELISAs (not shown). The increase in EBV-gp350/220 specific antibody appeared to plateau on day 84 after the initial boost, but then further increased after the second and third boosts. There was a significant difference in antibody titers of mice immunized with UV-EBV, compared with soluble gp350/220 ED and EBVgp350/220-F VLP, although the slopes of the response curves were similar. Gp350/220 specific antibody was absent from TNE-immunized mice. All gp350/220-based immunogens produced long-term gp350/220-specific responses, though mice immunized with native EBV (UV-EBV) maintained significantly higher titers of gp350/220 antibody compared to mice immunized with VLP or soluble recombinant protein.

### EBV-gp350/220 VLPs induce a dominant IgG1 subclass response

**Figure 5:**
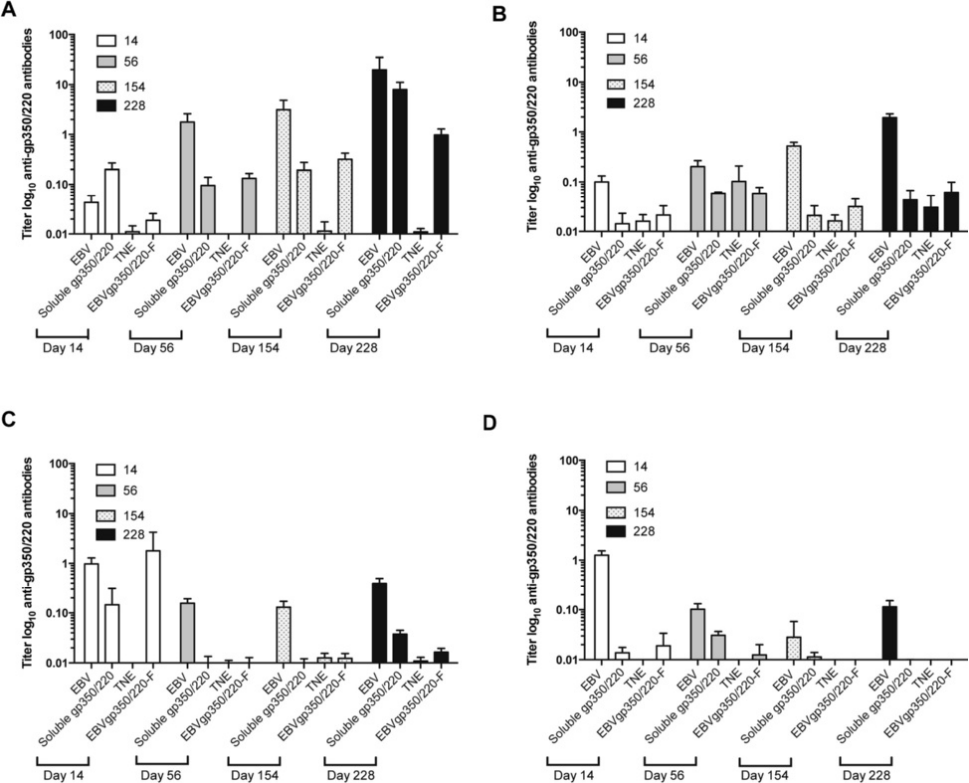
**Comparative analysis of IgG subclass responses to vaccination with EBVgp350/220 containing immunogens: EBVgp350/220-F VLPs, UV-EBV and soluble recombinant gp350/220 ED.** Serum IgG subclass titers were determined on days 14, 56, 154 and 228 post-vaccination by gp350/220 ED-based ELISA. Immunizations were carried out as described in Figure 4. TNE was the vehicle control. X-axis displays immunogen and date of vaccination. Y-axis indicates antibody titer specific for soluble recombinant gp350/320 ED on a log scale **(A)** IgG1 **(B)** IgG2a **(C)** IgG2b **(D)** IgG3.

To uncover the composition of IgG responses, we compared the subclass frequency of IgG antibodies induced by immunization with EBVgp350/220-F VLP, UV-EBV and recombinant soluble gp350/220 ED (Figure 5). IgG1 antibody, which typically reflects a T-helper type 2 response [38], was the most abundant subclass among mice irrespective of the gp350/220 immunogen. IgG1 responses uniformly increased over time when analyzed on days 14, 56, 154 and 228 post-immunization (Figure 5A). In contrast, titers of IgG2a, IgG2b and IgG3 did not significantly change or diminished over time. While IgG2a titers also appeared to trend upwards among mice immunized with UV-EBV (Figure 5B), (though to a 10-fold lesser degree than IgG1 titer) coordinate detection of IgG2a responses among the TNE-immunized control group in this assay alone (compare 5A, C, D) raised concern that the specificity of this reagent was compromised. A transient acute rise in IgG2b titers that accompanied primary chimeric VLP immunization did not persist.

### Anti EBV-gp350/220 antibodies generated following VLP immunization neutralize EBV infection *in vitro*

**Figure 6:**
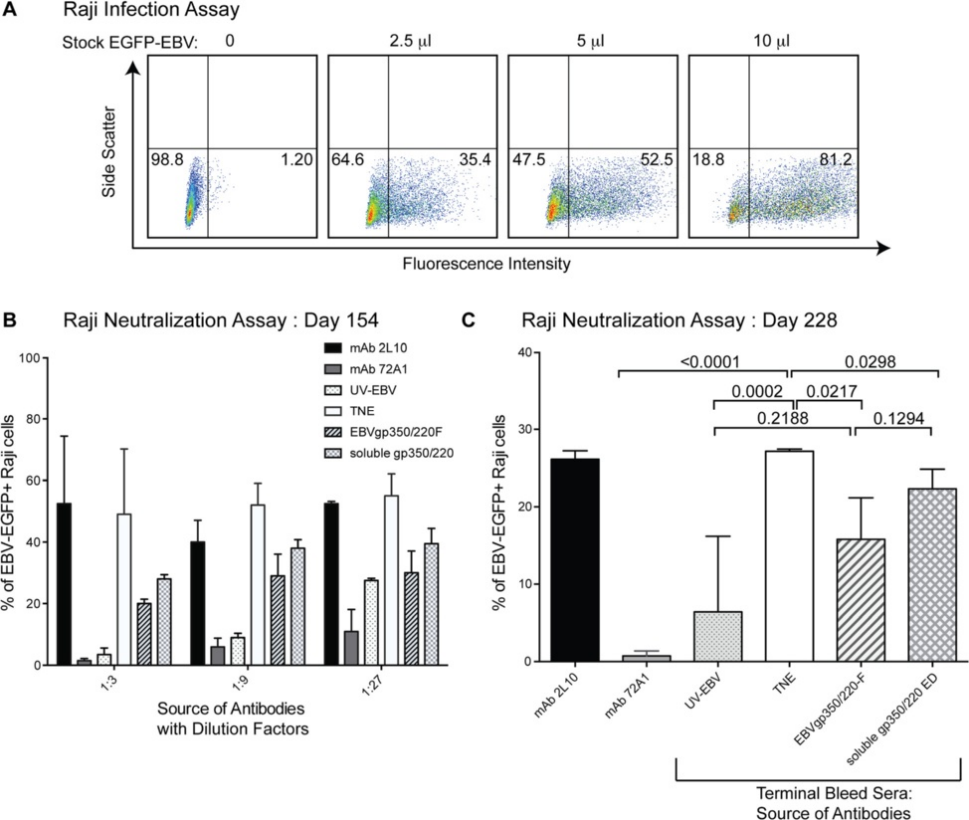
**Neutralization of Raji cell infection upon pre-incubation with antibodies generated following EBV-VLP immunization. (A)** Infection assay. Because EBV does not plaque, infectivity of EGFP-EBV from a frozen stock was directly quantitated by cytometry. Ten microliters stock virus in the absence of serum yielded ~80% infection (green fluorescence) 73 h after infection of Raji and was selected for neutralization experiments performed 111 days after the initial boost. Five microliters stock virus without serum yielded ~50% infection (green fluorescence) 73 h after infection of Raji and was used for terminal neutralization experiments. **(B)** Day 111 (long-term) Neutralization Assay. Pooled sera from groups of five BALB/c mice immunized with EBVgp350/220-F VLP, UV-EBV, soluble recombinant gp350/220 ED or TNE (vehicle control) were serially diluted as indicated and then pre-incubated with EGFP-EBV to assess neutralization (Methods). Infected cells were incubated at 37°C for 72 h at which time EGFP+ Raji cells were enumerated by cytometry. X-axis, neutralizing antibody source and dilution; Y- axis, percent EGFP+ Raji cells post-infection. **(C)** Terminal Neutralization assay. Pooled terminal bleed sera from groups of five BALB/c mice immunized with EBVgp350/220-F VLP, UV-EBV or soluble recombinant gp350/220 ED were pre-incubated with EGFP-EBV to assess neutralization (Methods). Infected cells were incubated at 37°C for 72 h at which time EGFP+ Raji cells were enumerated by cytometry. X-axis, neutralizing antibody source; Y-axis, percent EGFP+ Raji cells post-infection. Terminal sera or mAb controls were pre-incubated 1:1 with EGFP-EBV causing further virus dilution such that ~25% of Raji cells were maximally infected (fluoresced green) when pre-incubated with non-blocking mAb-2 L10 or TNE. Pre-incubation with mAb-72A1 (neutralizing) served as the positive control. Results are expressed as mean ± standard deviations (SD). Horizontal black lines terminating in short vertical lines compare sets of neutralization experiments with 2-sided p-values indicated above the line.

It is well known that titer is not the sole gauge of a protective antibody response, as certain immunogens can induce antibodies that promote, rather than block infection and high affinity blocking antibodies can be highly effective at low titers [39]. To evaluate the ability of antibodies generated in response to chimeric EBV-VLP, UV-EBV and soluble recombinant gp350/220 ED to block infection, we assessed the *in vitro* neutralizing antibody titers of sera collected (1) on day 154 (111 days after a single boost) and (2) on day 228 (terminal bleed collected 10 days after a fourth boost). Because EBV does not plaque and large virus quantities are difficult to obtain, EBV was first titered by the Raji cell infection assay (Figure 6A). Different amounts of a frozen stock of EGFP-EBV (0, 2.5, 5, 10 microliters) were incubated with Raji in the absence of serum. After infection with 10 μl of stock EGFP-EBV ‵80% of Raji cells reproducibly fluoresced upon detection by cytometry whereas 5 μl of stock EGFP-EBV reproducibly resulted in ~ 50% fluorescence (Figure 6A). When 10 μl EGFP-EBV was pre-incubated 1:1 with serial dilutions (1:3, 1:9, 1:27) of non-neutralizing mAb-2 L10 or TNE control sera fluorescence dropped to ~50% of infected Raji cells (Figure 6B). In contrast when serially diluted sera from mice immunized with EBVgp350/220-F VLPs, UV-EBV or soluble recombinant gp350/220 ED was likewise pre-incubated with EGFP-EBV, infection was significantly reduced in a dose dependent manner compared to TNE-immunized sera (Figure 6B). Purified mAb-72A1 (positive control) containing only IgG1 antibody directed to the gp350/220 attachment epitope was most effective at neutralization. Use of serial dilutions confirmed the specificity of the neutralizing antibody response.

In a second set of experiments neutralizing antibody was measured at the time of sacrifice ten days after a pre-terminal boost. When 5 μl EGFP-EBV was pre-incubated 1:1 with irrelevant mouse serum, further diluting virus, fluorescence dropped to ~25% of infected Raji cells (not shown). As predicted, pre-incubation of EGFP-EBV 1:1 with serum from TNE treated mice (negative control) produced ~ 27% fluorescence of Raji (Figure 6C) as did pre-treatment with the non-neutralizing anti-gp350/220 mAb-2 L10. In contrast when terminal sera from mice immunized with EBVgp350/220-F VLPs or UV-EBV was pre-incubated with EGFP-EBV, infection was reduced in comparison with TNE-immunized sera: 15% (p = 0.0217) and 5% fluorescent cells (p = 0.0002) (Figure 6C) respectively. As expected, purified mAb-72A1 (positive control) was most effective at neutralization (1% fluorescent cells, p = <0.0001 compared with TNE). Antibodies generated after immunization with soluble recombinant gp350/220 ED were least effective (22% fluorescent cells, p = 0.0298 versus TNE). Though the numbers are small, the comparative ability of antibodies generated in response to immunization with chimeric VLP versus UV-EBV to neutralize EBV infection of Raji cells was not significant (p = 0.2188).

## Discussion

Based on emerging evidence of the spectrum of EBV-associated diseases, Anthony Epstein first made a call for development of an EBV vaccine in 1976 [40]. To date, there is no licensed vaccine that prevents infection, AIM or any of the EBV-associated cancers. Because of the oncogenic potential of EBV, options for developing a safe, prophylactic and/or therapeutic EBV vaccine remain challenging. Similar to HSV1 replication defective vaccine candidates [41], EBV DNA packaging mutants [21] and disabled virions that lack the major oncoproteins [22] have appeal because of the multiplicity of potential immunogens present. However, incomplete knowledge of virion protein functions, concern about inadvertent association of oncogenic DNA/RNA fragments with assembled virions, limited production and release of virus, as well as current requirements for propagation in transformed human cell lines suggest such candidates may prove unable to meet stringent FDA safety guidelines. Additional concerns have been raised about EBV proteins expressed from replicating virus vectors such as Vaccinia or attenuated Measles [42] - the advantage and disadvantage being propagation of EBV-encoded nucleic acids and protein. Although vaccinia-based introduction of EBV vaccine targets, especially potential oncoproteins may be appropriate in patients with incurable tumors [43], current recombinant vaccinia vaccines are regarded as high risk because of frequent side effects even among healthy individuals. EBV capsids assembled from recombinant proteins expressed in insect cells have also been described as a potential vaccine candidate and would present repetitive antigen to the immune system [44]. However, at present their safety and their efficacy as neutralization targets remains to be documented.

EBVgp350/220 is the most abundant glycoprotein on the virus envelope and is the primary target of neutralizing antibodies following natural infection. Gp350/220 has long been a major focus of vaccine trials [16]. To date, vaccinia viruses expressing soluble recombinant gp350/220, attenuated measles virus expressing soluble recombinant gp350/220, truncated tetramers of the gp350/220 ED [45] as well as purified soluble gp350 protein have been evaluated to varying degrees either in animal models or in humans. However, controlled human Phase I/II clinical trials in which the immunogen was adequately characterized and in which efficacy could be statistically assessed remains limited. A three-dose regimen of purified soluble recombinant gp350 purified from CHO cell supernatants together with adjuvant was safe and immunogenic in healthy adults 18–25 years old residing in Europe [46], and in children scheduled for renal transplant [47]. Immunization induced gp350 specific neutralizing antibodies in the young adult group and provided protection from symptoms of AIM, however, EBV infection was not prevented [17]. The overall variable success of recombinant soluble protein trials underscores the historical observation that protein subunit vaccines generally display limited immunogenicity [18,19], and are not optimal for eliciting long-term protection. We reasoned that more physiologic presentation of gp350/220 to the immune system as would occur on exposure to the repetitive units of gp350/220 displayed on the virion envelope might achieve robust protective humoral as well as cell-mediated responses.

In this study we described development of a candidate chimeric EBV VLP vaccine with the potential to satisfy safety requirements and overcome limited immunogenicity. The synthesis, assembly, biological properties and humoral immune responses to this immunogen were characterized. First we showed that following transfection of cDNA encoding the chimeric glycoprotein, the gp350/220 ED was well expressed on the surface of diverse cell lines including CHO, 293, Vero and ELL-0 (a prerequisite for VLP assembly). Next we documented that after co-transfection of relevant cDNAs, VLPs assembled and were efficiently released into CHO supernatants. Demonstration of production in CHO was significant, as over two-thirds of recombinant therapeutic agents on the market are generated in CHO cell lines [48] that grow to high density in suspension and without FBS. Furthermore, no adventitious viral product, a major concern to regulatory authorities has been reported for CHO cells, in contrast to cell lines of human origin [49].

Subsequent analysis of the gradient purified chimeric VLPs by silver stain, immunoblot, and EM revealed that they were intact, pure and that the gp350/220 ED could be detected by distinct anti-gp350/220 mAbs. This suggested that the conformation of the attachment site was conserved and that some more distal portions of the ED were likely physiologically folded. The binding mechanisms used by native virus appeared to be retained as chimeric VLPs bound cells expressing the attachment receptors CD21 and CD35 and co-localized with each of these receptors. EM further revealed that chimeric VLPs resembled native EBV in their morphology and diameter, and immunogold staining revealed gp350/220 protein on the VLP surface.

EBV is a strictly human virus. Lack of an EBV vaccine is in part due to the paucity of robust *in vivo* models for studying EBV pathogenesis [50,51]. To begin to investigate the immunogenicity of EBVgp350/220-F VLPs, with a goal towards establishing efficacy in models such as humanized rodents, we immunized BALB/c mice with VLPs, UV-EBV or soluble recombinant gp350/220. No adjuvant was used in any of these experiments. We determined both the specific antibody titer and the ability of the antibodies to neutralize EBV infection *in vitro*. Total IgG anti-gp350/220 in sera of chimeric VLPs, UV-EBV and soluble recombinant gp350/220 slowly increased over time after primary immunization until day 43 at which time a first booster was administered. Following the boost, antibody titers rapidly rose among animals immunized with gp350/220 protein, peaking at day 56. No gp350/220 specific IgG antibody was detected in control mice (TNE). All anti-gp350/220 immunized mice developed long-term neutralizing antibody responses that were present 111 days after a single boost and were detected following additional boosts at the time of sacrifice on day 228.

Although antibody titers and virus neutralization was highest among groups of mice immunized with UV-EBV compared to EBVgp350/220-F VLP, several factors may account for this observation and point to specific methods for optimizing chimeric VLP immunogenicity. Whereas the equivalence of total gp350/220 protein content was established in the three experimental groups prior to immunization, UV-EBV was less highly purified than the chimeric VLPs raising the possibility of low-level contamination with environmental adjuvants that enhanced antibody responses. The potential contribution of distinct adjuvants to VLP immunogenicity will be assessed in future experiments. A unique density or distribution of gp350 on the native virus particle may enhance its immunogenicity. Whereas UV-EBV (and the soluble recombinant protein) were produced in primate cells, the chimeric VLP was produced in rodent cells, thus contamination with xenogeneic antigen may have further contributed to the enhanced immunogenicity of UV-EBV; and the soluble recombinant protein may de facto be less immunogenic than was scored herein. Furthermore antibody titers were detected using ELISA plates coated with primate (293-derived) recombinant gp350/220 – potentially amplifying the response of mice immunized with UV-virus and recombinant protein on this basis alone. We note that rather than enhancing immunogenicity as hypothesized, inclusion of the HR2 trimerization domain from NDV-F in the ED of the chimera may have contributed to some degree of misfolding and actually blunted the immune response to the chimeric VLP – this is currently being addressed. The quantity of gp350/220 ED expressed on the surface of VLPs can potentially be manipulated to introduce more immunogen in a more compact form that may enhance immunogenicity, or simple addition of more chimeric VLPs may enhance neutralizing antibody responses. Analogous to experiments conducted with respiratory syncytial virus [35] a further strength of this NDV platform is the potential to accommodate other EBV proteins able to block entry or initiation of infection upon introduction into a single VLP or by immunization with a combination of VLPs bearing different EBV chimeras, similar to VLP strategies that prevent human papillomavirus infection and associated cancers [52,53].

## Conclusions

While many acute viral infections and two major virus-associated malignancies have been reduced or eliminated through vaccination, adverse consequences resulting from EBV infection continue to emerge. Paradoxically, global development leading to delayed primary EBV infection has increased the incidence of AIM and with it a predisposition to Hodgkin lymphoma and autoimmune disease. In addition to prevention of EBV+ endemic malignancies, increasing survival of a spectrum of immunocompromised hosts susceptible to EBV+ lymphoproliferative diseases (e.g. the elderly, transplant recipients, persons with HIV or advanced autoimmune diseases) highlights a critical need for an effective immunogen – that is an EBV vaccine that is safe, prevents infection and/or limits the manifestations of disease. Herein we show that a simple innovative NDV-VLP platform supports generation of abundant chimeric EBVgp350/220-F VLPs devoid of oncogenic potential. These VLPs are efficiently produced, released, purified and quantified from CHO cells, a preferred repository for human biologics. The VLPs are nontoxic and stimulate production of long-lasting and neutralizing antibodies in standard BALB/c mice. Particle optimization and broad evaluation of immunologic properties in additional animal models merit ongoing investigation of this novel EBV vaccine candidate.
